# Assessing performance of directed functional connectivity measures in the presence of common source

**DOI:** 10.1186/1471-2202-16-S1-P124

**Published:** 2015-12-18

**Authors:** Jisung Wang, Heonsoo Lee, Seunghwan Kim

**Affiliations:** 1 Physics Department, Pohang University of Science and Technology, Pohang, South Korea

## 

The mixing problem of electroencephalography (EEG) in the presence of common source could affect directed functional connectivity measures, resulting in an incorrect directionality of information flow between two signals. Here we introduce directed Weighted Phase Lag Index (dWPLI), a signed version of Weighted Phase Lag Index (WPLI) and compare this measure to Granger Causality (GC), Symbolic Transfer Entropy (STE), Phase Slope Index (PSI), and directed Phase Lag Index (dPLI) under common source effects. Robustness of the measures was tested in both analytic and simulating ways.

For the simulated time series, signals were generated from unidirectionally coupled autoregressive model and linearly mixed to achieve volume conduction and common noise effects.

**Figure 1:**
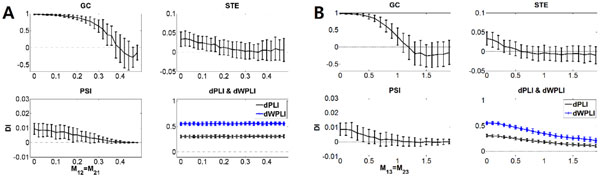
**A. Directionalities of five directed connectivity measures under the volume conduction effect**. Positive Directionality Index (DI) implies correct directionality of information flow. As M (mixing ratio) increases, the DIs of GC, STE and PSI decrease. However, as expected in analytic calculation, the dPLI and dWPLI are not affected. (DI is defined as a normalized information flow.) **B**. Directionalities of five directed connectivity measures under the common noise effect. As M (noise-to-signal ratio) increases, the DIs' signs of GC, STE and PSI change while those of dPLI and dWPLI do not.

As expected from the analytic calculation, dPLI and dWPLI were unaffected by the volume conduction while PSI, GC and STE were largely affected. (Figure 1A). The mean percentages of false identification were 14.00±7.66, 22.35±4.78, 27.38±7.04, 1.99±1.36, and 1.63±1.28, for the GC, STE, PSI, dPLI, and dWPLI, respectively.

For the common noise case, only dPLI and dWPLI had their signs preserved. (Figure 1B). The mean percentages of false identification were 30.14 ± 16.86, 49.74 ± 16.63, 34.72 ±10.23, 2.68 ± 1.90, and 2.34 ± 1.55.

Furthermore, the dWPLI outperformed dPLI for the common noise case (p <0.01, Wilcoxon signed-rank test) which was also predicted from analytic calculation.

Present study shows the common source effects might lead to biased results with incorrect directionality of information flow. Among the five directed functional connectivity measures, dWPLI is much less affected by common source effects.

